# Whole-Body Parametric Imaging of ^18^F-FDG PET Using uEXPLORER with Reduced Scanning Time

**DOI:** 10.2967/jnumed.120.261651

**Published:** 2022-04

**Authors:** Yaping Wu, Tao Feng, Yizhang Zhao, Tianyi Xu, Fangfang Fu, Zhun Huang, Nan Meng, Hongdi Li, Fengmin Shao, Meiyun Wang

**Affiliations:** 1 Department of Medical Imaging, Henan Provincial People’s Hospital, Henan, China;; 2 UIH America Inc., Houston, Texas; and; 3 United Imaging Healthcare, Shanghai, China

**Keywords:** image reconstruction, radiotracer tissue kinetics, dual injections, PET parametric imaging, reduced scanning time, total-body PET

## Abstract

Parametric imaging of the net influx rate (*K_i_*) in ^18^F-FDG PET has been shown to provide improved quantification and specificity for cancer detection compared with SUV imaging. Current methods of generating parametric images usually require a long dynamic scanning time. With the recently developed uEXPLORER scanner, a dramatic increase in sensitivity has reduced the noise in dynamic imaging, making it more robust to use a nonlinear estimation method and flexible protocols. In this work, we explored 2 new possible protocols besides the standard 60-min one for the possibility of reducing scanning time for *K_i_* imaging. **Methods:** The gold standard protocol (protocol 1) was conventional dynamic scanning with a 60-min scanning time. The first proposed protocol (protocol 2) included 2 scanning periods: 0–4 min and 54–60 min after injection. The second proposed protocol (protocol 3) consisted of a single scanning period from 50 to 60 min after injection, with a second injection applied at 56 min. The 2 new protocols were simulated from the 60-min standard scans. A hybrid input function combining the population-based input function and the image-derived input function (IDIF) was used. The results were also compared with the IDIF acquired from protocol 1. A previously developed maximum-likelihood approach was used to estimate the *K_i_* images. In total, 7 cancer patients imaged using the uEXPLORER scanner were enrolled in this study. Lesions were identified from the patient data, and the lesion *K_i_* values were compared among the different protocols. **Results:** The acquired hybrid input function was comparable in shape to the IDIF for each patient. The average difference in area under the curve was about 3%, suggesting good quantitative accuracy. The visual difference between the *K_i_* images generated using IDIF and those generated using the hybrid input function was also minimal. The acquired *K_i_* images using different protocols were visually comparable. The average *K_i_* difference in the lesions was 2.8% ± 2.1% for protocol 2 and 1% ± 2.2% for protocol 3. **Conclusion:** The results suggest that it is possible to acquire *K_i_* images using the nonlinear estimation approach with a much-reduced scanning time. Between the 2 new protocols, the protocol with dual injection shows the greatest promise in terms of practicality.

## Visual Abstract

**Figure:**
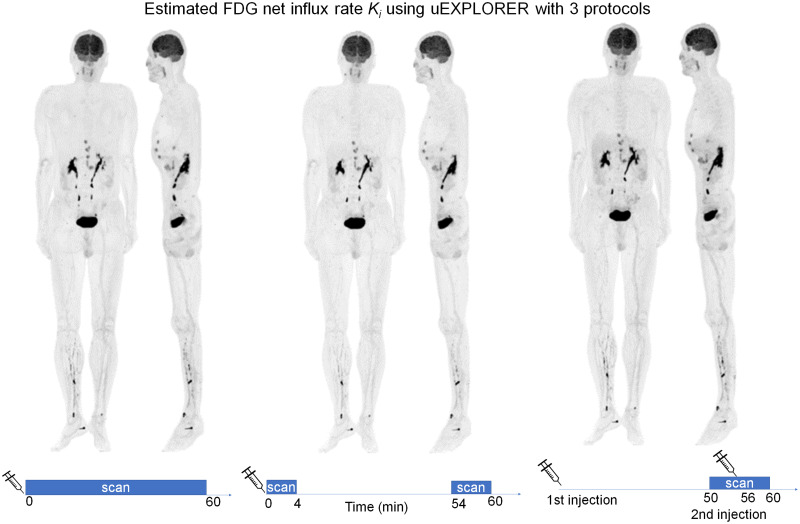

PET with SUVs (*1*,*2*) is widely used in clinical oncology for tumor imaging. However, the use of SUVs suffers from several drawbacks (*3*). For instance, the kinetics of uptake time for ^18^F-FDG may vary significantly in different tissues (*4*). In addition, the use of SUV measurements to differentiate malignant tumors from processes such as inflammation is challenging (*5*–*7*).

Parametric imaging provides an alternative to SUV imaging and has the potential to provide added information. For ^18^F-FDG studies, a few parameters are commonly derived, such as the net influx rate (*K_i_*), the delivery rate constant (*K*_1_), and blood fractions in tissue. *K_i_* is more commonly used and often acquired using graphical methods because of its simplicity (*8*). The acquired *K_i_* has been found to yield improved specificity at a similar sensitivity for cancer detection (*9*). *K_i_* images have also been found to yield better results for tumor volume delineation than SUV images (*10*). ^18^F-FDG *K*_1_ alone or combined with *K_i_* was found to be an indicator of tumor subgroup (*11*) and a way to evaluate chemotherapy response (*12*). The combined ^18^F-FDG parameters were found to be helpful for assessing metabolic tumors (*13*) as well.

Compared with SUV imaging, parametric imaging also has its challenges. One is the need for an accurate input function. The conventional approach requires invasive sampling of arterial blood. In recent years, more studies have been suggesting that the image-derived input function (IDIF) (*14*,*15*), population-based input function (*16*), or hybrid input function with both image data and population samples (*17*) can be used as a noninvasive replacement. Another practical issue is the much-increased scanning time. In estimating *K_i_* using the conventional Patlak method, a much longer scan is unavoidable. This is because the *K_i_* image is the slope image in the Patlak model; with slow-changing dynamics, it requires a long scanning time to accurately estimate the change in activity. As a result, a minimal scanning time of 30 min is often used to estimate *K_i_* with the Patlak model. Compared with state-of-art whole-body SUV scans, which last less than 10 min, the much-increased scanning time has limited the daily application of parametric imaging. The much-increased scanning time also increases the likelihood of patient motion during scans, which may further degrade image quality.

Although a single-bed-position acquisition is usually conventional for parametric imaging, whole-body Patlak analysis using regular scanners (*18*) or the total-body uEXPLORER scanner (*19*) has recently been proposed and validated. Whole-body parametric imaging provides a unique opportunity for the inspection of disseminated disease—also a major application of PET imaging.

Compared with the graphical method, *K_i_* can also be estimated using a nonlinear approach with an 2-tissue-compartment irreversible model. An entire time–activity curve consisting of a 60-min scan or an even longer scanning time is usually used for this purpose. With the uEXPLORER (*20*), the much-increased sensitivity of the whole-body scan has dramatically reduced noise in the reconstructed dynamic images. This reduction has made nonlinear estimation more robust. An advantage of nonlinear estimation is that it can better use dynamic data than models (Patlak model) that require data after equilibrium for estimation (*21*), therefore providing more freedom in protocol design. In previous studies, we demonstrated the possibility of reducing scanning time for estimating parameters such as *K*_1_ and the blood fraction (*22*). In this work, we further explored the possibility of accurately estimating *K_i_* using a much shorter dynamic scanning sequence with a total scanning time of 10 min for whole-body imaging. Two alternatives were investigated. One used a combination of early-time-point and late-time-point scanning (dual-time-point scanning), and the other used a dual-injection protocol to combine both early dynamic information and late dynamic information within a single scan.

## MATERIALS AND METHODS

### Scanning Protocols

Three protocols were studied in this work. Protocol 1 was a conventional 0 min to 60 min dynamic scan. It was used as the gold standard to evaluate the performance of the 2 proposed protocols.

The first proposed protocol (protocol 2) consisted of a combination of 2 time points, that is, an early time point at 0–4 min after injection and a late time point at 54–60 min after injection. To minimize use of scanner time, the patients were scanned twice, with registration taking place between the scans. As a proof of concept, this protocol was simulated by excluding the 4 min to 54 min postinjection interval of an entire 0 min to 60 min dynamic scan. The main goal of this protocol was to examine the accuracy of the estimation by using information from only the early and late phases.

**FIGURE 1.**
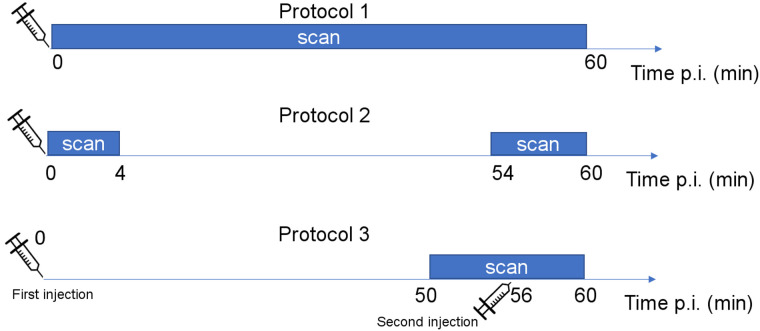
Illustration of the 3 protocols proposed in this study. p.i. = after injection.

The second proposed protocol (protocol 3) used a single scanning period of 50–60 min after injection, with the help of a dual-injection scheme. The first injection occurred at t = 0 (protocol began with the first injection), and the second injection occurred 56 min later. In this case, the last 4 min provided the early dynamic data, and the first 6 min provided information similar to that of the second scanning period in protocol 2. This scanning protocol was simulated by combining the dynamic images from 0 to 4 min after injection with those from 56 to 60 min after injection. Figure 1 illustrates the 3 protocols, and Table 1 shows their dynamic time frames.

**TABLE 1:** Dynamic Frames for Different Protocols

Parameter	Protocol 1	Protocol 2	Protocol 3
Start time (min)	0	0	50
Dynamic frames	5 (s) × 30	5 (s) × 30	120 (s) × 3
	30 (s) × 15	30 (s) × 3	5 (s) × 30
	120 (s) × 25	50 (min) × 1 (no scan); 120 (s) × 3	30 (s) × 3

### Input Functions

For protocol 1, the IDIF was used. The ascending aorta was used to extract the IDIF, as it is less affected by respiratory motion. For the other 2 protocols, with the limited scanning time, the IDIF was not available for the entire dynamic range. In this work, we acquired the input function using a hybrid approach by combining the population-based input function, the model-based input function, and the IDIF. The input function for protocol 2 is proposed as

$$C_{p}(t)≜\{\begin{array}{l} C_{\text{image}\;1}\left(t\right)\\ \begin{array}{l} \text{μ}e^{-\text{γ}(t-t_{1})}C_{p0}\left(t\right)\;\\ C_{\text{image}\;2}\left(\text{t}\right)\; \end{array} \end{array}\;\;\;\;\;\;\;\;\;\begin{array}{r} t\leq t_{1}\\ t\leq t_{1}\;\text{and}\;t\leq t_{2}\\ t\leq t_{2} \end{array}$$ Eq. 1

where $C_{\text{image}\;1}\left(t\right)$ is the IDIF of the first 4 min and $C_{\text{image}\;2}\left(t\right)$ is the IDIF of the last 6 min. $C_{p0}\left(t\right)$ is the population-based input function, γ and μ are the scaling constants that satisfy $\text{μ}C_{p0}\left(t_{1}\right)=C_{\text{image}\;1}\left(t_{1}\right),\;$ and $\text{μ}e^{-\text{γ}(t_{2}-t_{1})}C_{p0}\left(t_{2}\right)=C_{\text{image}\;2}\left(t_{2}\right)$.

For protocol 3, the input function was based on 2 assumptions: the first is that the later phase of the input function can be approximated as a single exponential function, and the second is that the shape of the input function from the second injection has the same shape as the input function from the first injection. In literature studies with multiple injections in 1 patient, the similarity of the produced input function (*23*) supports the second assumption.

The IDIF was first separated into 2 regions: before the second injection (*C_B_*(*t*)) and after the second injection (*C_A_*(*t*)), where *t*_0_ represents the second injection time. An exponential curve ($C_{B0}e^{-bt}$) was used to fit *C_B_*(*t*), and the contribution of the second injection was estimated by subtracting the exponential curve from the IDIF *C_A_*(*t*), that is, *C_A_*(*t*) *−* $\;C_{B0}e^{-bt}$. The contribution from the second injection was treated as the early-phase input function ($C_{\text{image}\;1}\left(t\right)$), like that in protocol 2, and *C_B_*(*t*) was treated as the late phase input function ($C_{\text{image}\;2}\left(t\right)$), like that in protocol 2. The missing part was approximated using the same approach as shown in Equation 1.

For both protocol 2 and protocol 3, the original IDIF acquired using the whole dynamic process was used as the gold standard.

### Maximum-Likelihood Estimation

The dynamic changes in ^18^F-FDG within the human body can be approximated using the 2-tissue-compartment model, where the first compartment (*C*_1_) describes perfusion of ^18^F-FDG to the tissue and the second compartment (*C*_2_) models the phosphorylation process within the cells. The 2 compartments can be modeled mathematically using the rate constants

$$\{\begin{array}{c} \frac{dC_{1}}{dt}=K_{1}C_{p}+k_{4}C_{2}-k_{2}C_{1}-k_{3}C_{1}\\ \frac{dC_{2}}{dt}=k_{3}C_{1}-k_{4}C_{2} \end{array}$$ Eq. 2

where *K*_1_ and *k*_2_ describe the forward and backward perfusion process of ^18^F-FDG in the tissue, and *k_3_* and *k_4_* describe the phosphorylation and dephosphorylation process. In many cancer cells, FDG-6-phosphate is only minimally dephosphorylated and is trapped within the cell (*24*). This process allows us to simplify the model by assigning a value of 0 to *k*_4_. The acquired dynamic PET image, X(t), can be represented using the equation below when *k*_4_ is 0.

$$X=v_{b}C_{p}+C_{1}+C_{2}\;=v_{b}C_{p}+\frac{k_{2}K_{1}}{k_{2}+k_{3}}\text{exp}\left(-(k_{2}+k_{3})t\right)⊗C_{p}\left(t\right)+\frac{k_{3}K_{1}}{k_{2}+k_{3}}\int^{\text{​}}\;\;C_{p}dt$$ Eq. 3

where $K_{1}^{\prime }=K_{1}\frac{k_{2}K_{1}}{k_{2}+k_{3}}$, $k_{2}^{\prime }=k_{2}+k_{3}$, $K_{i}=\frac{k_{3}K_{1}}{k_{2}+k_{3}}$, and $C_{i}\left(t\right)=\int^{\text{​}}C_{p}dt$. With these definitions, the above equation can be written as

$$\;X=v_{b}C_{p}+C_{1}+C_{2}\;=v_{b}C_{p}(t)+K_{1}^{\prime }\text{exp}\left(-k_{2}^{\prime }t\right)⊗C_{p}\left(t\right)+K_{i}C_{i}\left(t\right)$$ Eq. 4

The above equation is similar to the Patlak model, where the *K_i_* has the same definition as that in the Patlak model, and the combined effect of $v_{b}+K_{1}^{\prime }\text{exp}$ $\left(-k_{2}^{\prime }t\right)⊗/C_{b}(t)$ was treated as a constant after equilibrium in the Patlak model.

With the assumption that the voxel values in the dynamic image approximately follow a scaled Poisson distribution (*25*), the maximum-likelihood estimation approach was used for estimating $K_{i}$. The update equation for $K_{i}$ can be derived as

$$K_{i}^{p+1}=\frac{K_{i}^{p}}{\sum_{t}C_{i}\left(t\right)}\underset{t}{\sum }\frac{C_{i}\left(t\right)X\left(t\right)}{{\hat{C}}^{p}\left(t\right)}$$ Eq. 5

where ${\hat{C}}^{p}\left(t\right)={v\;}_{b}^{p}C_{b}+K\prime_{1}^{p}e^{-k\prime_{2}^{p}t}⊗C_{p}\left(t\right)+K\prime_{i}^{p}C_{i}\left(t\right)$ is the estimated dynamic image at time *t* given the estimated parametric images and *p* is the iteration number. The effects of different frame lengths were included in *C_p_* and *C_i_* in the above equations. The derivation is similar to our previous proposed update equation for $v_{b}$ and $K_{1}$ (*22*). For whole-body imaging, the input function $C_{p}$ may also be subject to the delay and dispersion effects. The delay effect is modeled and estimated using the same approach as one previously proposed (*22*). In total, 5 parameters, including *K*_1_′, *k*_2_′, *v_b_, K_i_*, and the time delay, were estimated jointly. The estimated *K_i_* was analyzed subsequently because it is the target of interest in this study.

### Patient Data and Image Reconstruction

In our study, 7 potential cancer patients (Table 2) referred to the Henan Hospital were imaged using the uEXPLORER scanner (Shanghai United Imaging Healthcare) with the dynamic scanning protocol. The patient group was preselected to exclude those with significant motion artifacts and those with nonbolus input functions. Visual examinations were used to determine the motion artifacts, and the exclusion criteria were examinations with visible motion greater than 5 voxels or 15 mm. The dynamic study was approved by the Institutional Review Board of the hospital, and written informed consent was obtained from each patient. The patients included 4 men and 3 women, with a weight of 66±13 kg and an injected dose of 273±60 MBq (mean ± SD). The leg was chosen as the injection site because it is closer to the end of the gantry.

**TABLE 2:** Patient Data Used in This Study

Patient no.	Sex	Weight (kg)	Injected dose (MBq)	Preliminary diagnosis
1	M	75	224.7	Prostate cancer
2	F	60	223.5	None
3	F	50	246.4	Pulmonary nodule
4	M	60	317.1	Space-occupying lesion (brain)
5	M	83	306.0	Gastric cancer
6	F	55	219.6	Leiomyoma
7	M	81	375.7	Pulmonary nodule

Dynamic images were reconstructed using the vendor-recommended settings with random, scatter, attenuation, normalization, and dead-time corrections; the reconstructed images had a 2.89-mm slice thickness and a 3.125-mm voxel size in the transaxial plane. The number of voxels in the reconstructed image was 192 by 192 by 672. Time-of-flight reconstructions were applied using manufacturer-supplied reconstruction software (ordered-subsets expectation maximization with 3 iterations and 24 subsets) with the point-spread-function model.

An alternate update approach was applied for the joint estimation process. Twenty-seven main iterations were used. In each iteration, 6 subiterations were used for *K_i_*, *K*_1_′, and the blood fraction (with a total iteration number of 162); 2 subiterations were used for the time delay; and 1 subiteration was used for *k*_2_′. The *K_i_* estimated using protocol 1 with the IDIF was selected as the gold standard. *K_i_* was also estimated using the conventional Patlak model for comparison. In the Patlak method, data from 20 min after injection to 60 min after injection with IDIF were used. The framing sequence was the same as for protocol 1. No postsmoothing filter was applied. The noise of the estimated images was calculated using VOIs in the thigh muscle region. The coefficient of variation was used as the surrogate for noise. A region-growing approach with a threshold of 90% of the maximum value in the *K_i_* image (protocol 1) was used for lesion acquisition. The average *K_i_* value of the lesions from images acquired using different methods was also measured to study quantitative accuracy.

## RESULTS

**FIGURE 2.**
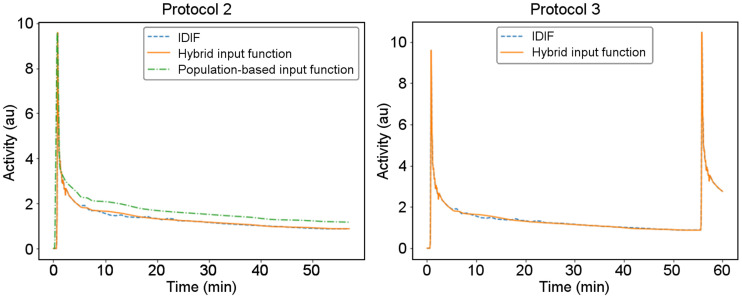
Comparison of IDIF and hybrid input function for protocols 2 and 3. Original population-based input function is also displayed for comparison. au = arbitrary units.

Figure 2 shows the input functions acquired from a patient using the IDIF approach and the proposed hybrid approach. The original population-based input function (*17*) is also displayed with normalized peak value. A significant difference existed between the population-based input function and the IDIF when normalized to the same peak value, but good agreement could be achieved with the hybrid method. For all patient data, the average area-under-the-curve ratio between the hybrid input function and the IDIF was 1.03 ± 0.04, suggesting it is possible to use the hybrid input function for *K_i_* estimation when the whole dynamic data are unavailable.

**FIGURE 3.**
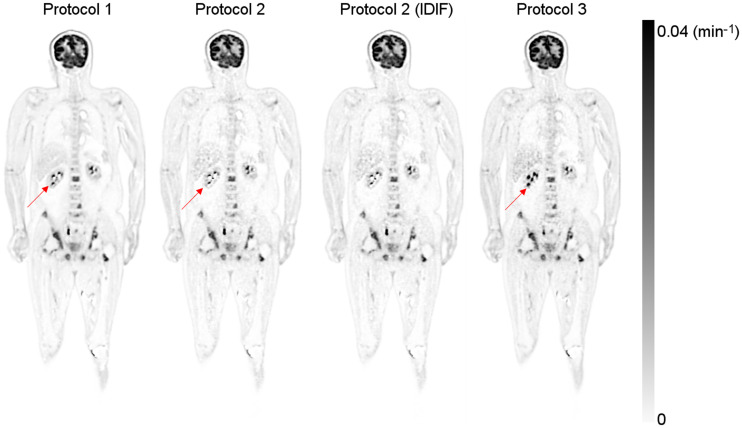
Estimated *K_i_* image of patient with prostate cancer. Arrows show regions with large *K_i_* differences using different protocols.

**FIGURE 4.**
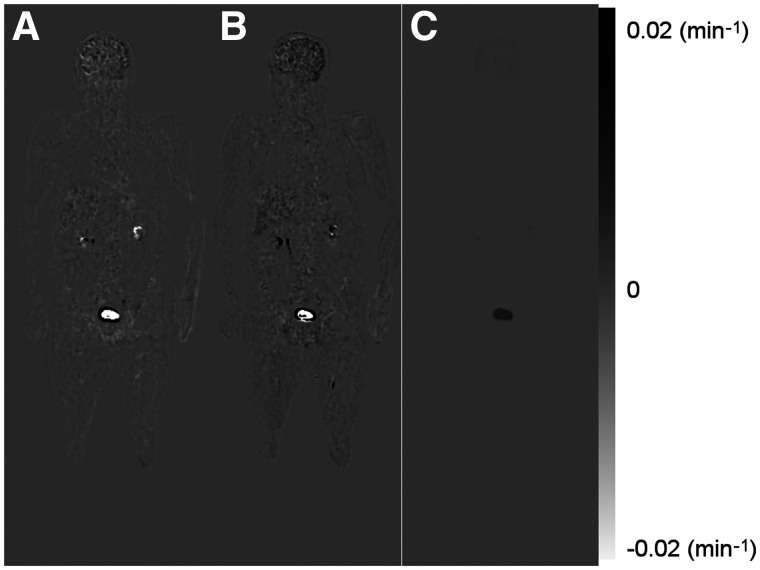
(A) Difference image of *K_i_* between protocols 2 and 1. (B) Difference image of *K_i_* between protocols 3 and 1. (C) Difference image of *K_i_* estimated using IDIF and hybrid input function with protocol 2.

**FIGURE 5.**
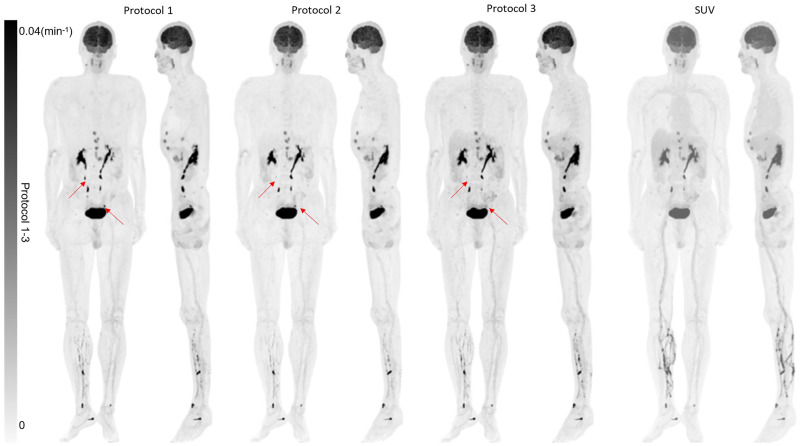
Maximum-intensity-projection PET image of *K_i_* from protocols 1–3 and SUV image acquired at 60 min. Arrows show regions with large *K_i_* differences using different protocols.

Figure 3 shows the reconstructed *K_i_* images of patient 1 using the 3 protocols (*K*_1_′ and *k*_2_′ images are included in Supplemental Figs. 1 and 2; supplemental materials are available at http://jnm.snmjournals.org). The same color scale was used for all images. The IDIF was used in protocol 1, and both the IDIF and the hybrid input function were used in the other protocols. The *K_i_* images acquired using protocols 2 and 3 were visually comparable to that using protocol 1 but noisier. Figure 4 shows the difference images of *K_i_* generated using different protocols (Supplemental Fig. 3 shows the corresponding percentage images). Minimal difference was observed between IDIF-based and hybrid input function–generated *K_i_* images. Figure 5 shows the maximum-intensity projections of the reconstructed *K_i_* images, as well as the SUV image for patient 5. The same scale was used for all *K_i_* images, and the SUV image was scaled for comparable muscle uptake.

Image noise using the 3 estimations was also calculated. The average coefficient of variation was 0.12 ± 0.04 for *K_i_* images estimated using protocol 1 in the thigh muscle region, 0.22 ± 0.05 for *K_i_* images estimated using protocol 2, and 0.20 ± 0.04 with protocol 3. The much-reduced noise level in protocol 1 was likely caused by the long scanning time. Protocol 3 also showed a reduced noise level when compared with protocol 2; this reduction was likely caused by the use of summed data, as it is effectively 2 times the dose compared with protocol 2.

**FIGURE 6.**
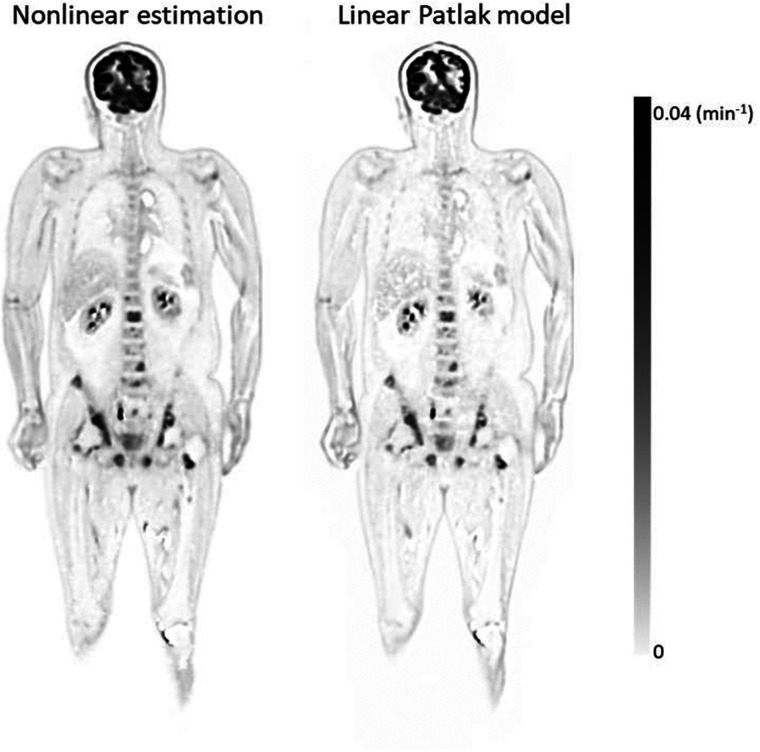
Estimated *K_i_* image using nonlinear model (protocol 1) and linear Patlak model.

Using protocol 1, *K_i_* was calculated with the proposed nonlinear approach and the conventional Patlak approach (Fig. 6). The images generally agree with each other, with some minor differences. The noise level in the nonlinear estimation was visually lower than that in the linear estimation, as agrees with literature findings (*26*). A higher muscle-background *K_i_* value was detected in the nonlinear approach.

**FIGURE 7.**
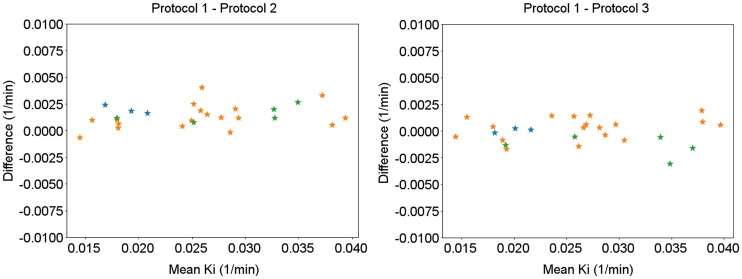
Bland–Altman plot for estimated *K_i_* in different lesions using different protocols. *x*-axis shows mean *K_i_,* and *y*-axis shows *K_i_* difference. Lesions from different patients are encoded using different colors.

In total, 26 lesions were identified and segmented from the patients. The same region of interest was used for different *K_i_* images generated in different protocols for consistency. The average diameter of the segmented lesions was 13.8 mm. The *K_i_* values inside the region of interest measured by the gold standard and the protocols with the reduced scanning time are plotted in Figure 7. An example of the fitted time–activity curve is included in Supplemental Figure 4. The mean difference between protocol 2 and protocol 1 was 2.8% ± 2.1%. The mean difference between protocol 3 and protocol 1 was 1% ± 2.2%. This result suggests that with a total scanning time of 10 min, the new protocols were able to maintain quantitative accuracy for the lesions despite the much-reduced scanning time.

## DISCUSSION

The *K_i_* difference between images (Fig. 4C) estimated using IDIF and the hybrid input function was negligible in most cases, suggesting that the hybrid input function can be a reliable approach for estimating *K_i_* images. However, with the hybrid input function, quantitative errors can still be present because of patient variations. The hybrid input function was also noninvasive and does not rely on data outside the acquisition period, making it easier to be incorporated into clinical studies.

Because of the residual activity around the injection site, some differences were present in the veins on the left leg among the different protocols. A large difference in *K_i_* was observed in the kidney region among the different protocols. The difference might be explained by the fact that the 2-tissue-compartment irreversible model cannot accurately model the renal excretion process, and therefore a large difference can be expected with different protocols. In general, *K_i_* images show much-improved lesion contrast when compared with SUV images, suggesting improved clinical value with *K_i_* imaging.

Although good quantitative accuracy was observed in the lesions, a slight overestimation was observed in the muscle and liver region with the nonlinear approach and protocol 3. One reason could be the reduction in estimation accuracy due to combined early-phase and late-phase information in the dynamic data. As shown in the supplemental figures, the accuracy of estimated *K*_1_′ and *k*_2_′ in protocol 3 was also not as good as that in protocol 2. Another reason could be the model mismatch effect. Because it was shown that the 2-tissue-compartment irreversible model may not be true in some tissues, as a nonzero *k*_4_ can be expected in some normal cells (*24*), different estimated results can be expected with different estimation methods or protocols.

A limitation of our approaches is that they require 2 scanning sessions (protocol 2) or 2 ^18^F-FDG administrations (protocol 3). This requirement makes the methods less practical but reduces the overall time spent on the PET/CT system. Protocol 2 requires additional image registration, which was not modeled in this study. The additional image registration may also introduce image artifacts that were not studied here. A second CT scan or a low-dose CT scan may also be required in the second scan for accurate image registration and attenuation correction. These challenges make protocol 2 less practical. On the other hand, the estimation method in protocol 2 provides a foundation for protocol 3 to work, as it shows that *K_i_* can be estimated by combining the early-phase and late-phase dynamic data. Protocol 3 provides a much better alternative for practical application of fast *K_i_* imaging with dual injections, as the data were acquired in a single scan frame without the need for registration or another CT scan. The absence of a second scan also makes patient management much easier and—because of the much shorter scanning time—reduces the likelihood of voluntary patient motion. However, patient motion may still impact the method, and therefore, motion compensation is still required for an improved quantitative result. There are potential challenges in protocol 3 as well. One is the assumption that the early-phase input function is the same as the second bolus injection. Future studies are required to study the impact of this effect. The direct addition of the images for simulating protocol 3 also doubles the effective injected dose, making the estimated noise in protocol 3 smaller than that in protocol 2. When the injected dose is kept the same, image noise is expected to be higher when using protocol 3.

van Sluis et al. also showed the possibility of reducing the scanning duration using the conventional Patlak model with the help of the population-based input function (*27*). The advantage of using the Patlak model is that it requires only a single injection. With the dual-injection protocols proposed in this study, we can achieve an even shorter scanning time and the potential for multiparametric imaging. Both approaches could be useful in clinical situations to promote the practical use of parametric imaging protocols.

## CONCLUSION

In this study, we have shown that with the modified protocols, it is possible to dramatically reduce the required scanning time for whole-body *K_i_* imaging to 10 min. The estimation of *K_i_* is possible because of the presence of both early-phase and late-phase information in the new protocols. The reduction in scanning time makes it easier to incorporate *K_i_* imaging into clinical routine.

## DISCLOSURE

This work was partially supported by the National Key R&D Program of China (2017YFE0103600), the National Science Foundation of China (81720108021), the Zhongyuan Thousand Talents Plan Project (ZYQ201810117), and the Zhengzhou Collaborative Innovation Major Project (20XTZX05015). Tao Feng and Hongdi Li are employed by UIH America, Inc., and Yizhang Zhao and Tianyi Xu are employed by United Imaging Healthcare. No other potential conflict of interest relevant to this article was reported.

## Notes

KEY POINTS **QUESTION:** Can whole-body *K_i_* imaging be achieved using a much-reduced scanning time (10 min)? **PERTINENT FINDINGS:** We have demonstrated with the new protocols (dual scanning points or dual injections) that it is possible to generate whole-body *K_i_* images with a total scanning time of 10 min. **IMPLICATIONS FOR PATIENT CARE:** A much-reduced scanning time for *K_i_* imaging improves the practicality for parametric imaging. A wider application of parametric imaging could be helpful for better diagnosis and treatment.

## References

[1] Delbeke D Coleman RE Guiberteau MJ. Procedure guideline for tumor imaging with ^18^F-FDG PET/CT 1.0. J Nucl Med. 2006;47:885–895. 16644760

[2] Lindholm P Minn H Leskinen-Kallio S Bergman J Ruotsalainen U Joensuu H. Influence of the blood glucose concentration on FDG uptake in cancer: a PET study. J Nucl Med. 1993;34:1–6. 8418248

[3] Adams MC Turkington TG Wilson JM Wong TZ. A systematic review of the factors affecting accuracy of SUV measurements. AJR. 2010;195:310–320. 20651185 10.2214/AJR.10.4923

[4] Shankar LK Hoffman JM Bacharach S. Consensus recommendations for the use of ^18^F-FDG PET as an indicator of therapeutic response in patients in National Cancer Institute Trials. J Nucl Med. 2006;47:1059–1066. 16741317

[5] Metser U Even-Sapir E. Increased 18F-fluorodeoxyglucose uptake in benign, nonphysiologic lesions found on whole-body positron emission tomography/computed tomography (PET/CT): accumulated data from four years of experience with PET/CT. In: *Seminars in Nuclear Medicine.* Vol 37. Elsevier; 2007:206–222. 10.1053/j.semnuclmed.2007.01.001 17418153

[6] Shreve PD Anzai Y Wahl RL. Pitfalls in oncologic diagnosis with FDG PET imaging: physiologic and benign variants. Radiographics. 1999;19:61–77. 9925392 10.1148/radiographics.19.1.g99ja0761

[7] Strauss LG. Fluorine-18 deoxyglucose and false-positive results: a major problem in the diagnostics of oncological patients. Eur J Nucl Med. 1996;23:1409–1415. 8781149 10.1007/BF01367602

[8] Patlak CS Blasberg RG. Graphical evaluation of blood-to-brain transfer constants from multiple-time uptake data: generalizations. J Cereb Blood Flow Metab. 1985;5:584–590. 4055928 10.1038/jcbfm.1985.87

[9] Magri A Krol A Lee W Lipson E McGraw W Feiglin D. A new method to determine probability of malignancy using dynamic breast F-18-FDG PET studies [abstract]. J Nucl Med. 2009;50(suppl 2):1445.

[10] Visser EP Philippens MEP Kienhorst L. Comparison of tumor volumes derived from glucose metabolic rate maps and SUV maps in dynamic ^18^F-FDG PET. J Nucl Med. 2008;49:892–898. 18483085 10.2967/jnumed.107.049585

[11] Sugawara Y Zasadny KR Grossman HB Francis IR Clarke MF Wahl RL. Germ cell tumor: differentiation of viable tumor, mature teratoma, and necrotic tissue with FDG PET and kinetic modeling. Radiology. 1999;211:249–256. 10189480 10.1148/radiology.211.1.r99ap16249

[12] Song SL Deng C Wen L. ^18^F-FDG PET/CT-related metabolic parameters and their value in early prediction of chemotherapy response in a VX2 tumor model. Nucl Med Biol. 2010;37:327–333. 20346872 10.1016/j.nucmedbio.2009.12.002

[13] Wang G Parikh M Nardo L. Total-body dynamic PET of metastatic cancer: first patient results [abstract]. J Nucl Med. 2020;61(suppl 1):208.

[14] Chen K Bandy D Reiman E. Noninvasive quantification of the cerebral metabolic rate for glucose using positron emission tomography, ^18^F-fluoro-2-deoxyglucose, the Patlak method, and an image-derived input function. J Cereb Blood Flow Metab. 1998;18:716–723. 9663501 10.1097/00004647-199807000-00002

[15] Feng T Tsui BMW Li X. Image‐derived and arterial blood sampled input functions for quantitative PET imaging of the angiotensin II subtype 1 receptor in the kidney. Med Phys. 2015;42:6736–6744. 26520763 10.1118/1.4934375 PMC4627933

[16] Rissanen E Tuisku J Luoto P. Automated reference region extraction and population-based input function for brain [^11^C] TMSX PET image analyses. J Cereb Blood Flow Metab. 2015;35:157–165. 25370856 10.1038/jcbfm.2014.194 PMC4294409

[17] Yao S Feng T Zhao Y. Simplified protocol for whole body Patlak parametric imaging with ^18^F‐FDG PET/CT: feasibility and error analysis. Med Phys. 2021;48:2160–2169. 32304095 10.1002/mp.14187

[18] Karakatsanis NA Lodge MA Tahari AK Zhou Y Wahl RL Rahmim A. Dynamic whole-body PET parametric imaging: I. Concept, acquisition protocol optimization and clinical application. Phys Med Biol. 2013;58:7391–7418. 24080962 10.1088/0031-9155/58/20/7391 PMC3941007

[19] Zhang X Xie Z Berg E. Total-body dynamic reconstruction and parametric imaging on the uEXPLORER. J Nucl Med. 2020;61:285–291. 31302637 10.2967/jnumed.119.230565 PMC8801950

[20] Badawi RD Shi H Hu P. First human imaging studies with the EXPLORER total-body PET scanner. J Nucl Med. 2019;60:299–303. 30733314 10.2967/jnumed.119.226498 PMC6424228

[21] Yu D-C Huang S-C Barrio JR Phelps ME. The assessment of the non-equilibrium effect in the ‘Patlak analysis’ of Fdopa PET studies. Phys Med Biol. 1995;40:1243–1254. 7568380 10.1088/0031-9155/40/7/007

[22] Feng T Zhao Y Shi H. Total-body quantitative parametric imaging of early kinetics of ^18^F-FDG. J Nucl Med. 2021;62:738–744. 32948679 10.2967/jnumed.119.238113 PMC8844261

[23] Lodge MA Jacene HA Pili R Wahl RL. Reproducibility of tumor blood flow quantification with ^15^O-water PET. J Nucl Med. 2008;49:1620–1627. 18832120 10.2967/jnumed.108.052076 PMC2587033

[24] Tsurusaki M Okada M Kuroda H Matsuki M Ishii K Murakami T. Clinical application of ^18^F-fluorodeoxyglucose positron emission tomography for assessment and evaluation after therapy for malignant hepatic tumor. J Gastroenterol. 2014;49:46–56. 23525980 10.1007/s00535-013-0790-5 PMC3895191

[25] Wilson DW Tsui BMW Barrett HH. Noise properties of the EM algorithm. II. Monte Carlo simulations. Phys Med Biol. 1994;39:847–871. 15552089 10.1088/0031-9155/39/5/005

[26] Zhang X Xie Z Wang G. Comparison of linear and non-linear total-body PET parametric imaging [abstract]. J Nucl Med. 2020;61(suppl 1):206.

[27] van Sluis J Yaqub M Brouwers AH Dierckx RAJO Noordzij W Boellaard R. Use of population input functions for reduced scan duration whole-body Patlak ^18^F-FDG PET imaging. EJNMMI Phys. 2021;8:11. 33547518 10.1186/s40658-021-00357-8 PMC7865035

